# Procalcitonin detection in human plasma specimens using a fast version of proximity extension assay

**DOI:** 10.1371/journal.pone.0281157

**Published:** 2023-02-16

**Authors:** Frederic Bedin, Vincent Benoit, Elsa Ferrazzi, Emeline Aufradet, Laurent Boulet, Agnes Rubens, Pascal Dalbon, Pierre Imbaud

**Affiliations:** 1 Innovation Department, BioMérieux SA, Marcy L’Etoile, France; 2 Assystem, Lyon, France; University of Helsinki: Helsingin Yliopisto, FINLAND

## Abstract

An exciting trend in clinical diagnostics is the development of easy-to-use, minimally invasive assays for screening and prevention of disease at the point of care. Proximity Extension Assay (PEA), an homogeneous, dual-recognition immunoassay, has proven to be sensitive, specific and convenient for detection or quantitation of one or multiple analytes in human plasma. In this paper, the PEA principle was applied to the detection of procalcitonin (PCT), a widely used biomarker for the identification of bacterial infection. A simple, short PEA protocol, with an assay time suitable for point-of-care diagnostics, is presented here as a proof of concept. Pairs of oligonucleotides and monoclonal antibodies were selected to generate tools specifically adapted to the development of an efficient PEA for PCT detection. The assay time was reduced by more than 13-fold compared to published versions of PEA, without significantly affecting assay performance. It was also demonstrated that T4 DNA polymerase could advantageously be replaced by other polymerases having strong 3’>5’ exonuclease activity. The sensitivity of this improved assay was determined to be about 0.1 ng/mL of PCT in plasma specimen. The potential use of such an assay in an integrated system for the low-plex detection of biomarkers in human specimen at the point of care was discussed.

## Introduction

There is a continuing need to develop easy to use, minimally invasive assays for disease screening, diagnosis and prevention. The PLA (Proximity Ligation Assay) and, more recently, the PEA (Proximity Extension Assay) can address these needs. PEA and PLA are homogeneous, dual-recognition immunoassays which allow for the sensitive and specific detection or quantitation of one or more analytes [1, 2]. Proximity assays rely on the principle of proximity probing, wherein an analyte is detected via the binding of at least two specific probes, which, when brought in close proximity one to the other after binding to the analyte, allow a nucleic acid-based signal to be generated and possibly quantified [3].

PEA was first described by Lundberg et al. in 2011 [1], for the detection of protein biomarkers in liquid samples. Several improvements were subsequently made with a focus on the potential of the technique for multiplex assays, as applied to the fields of proteomics and biomarker screening [4–6]. For each biomarker, a pair of antibodies conjugated to unique oligonucleotides (henceforth referred to as PEA probes or PEA conjugates) binds to their respective epitope. As a result, the probes are brought in close proximity one to the other and hybridize through the oligonucleotide 3’-ends, creating a short double-stranded template which is then extended through the subsequent addition of DNA polymerase. The DNA duplex thus formed can then be detected and quantified by real-time quantitative polymerase chain reaction (qPCR) with signal quantitatively proportional to the initial concentration of target protein in the sample. PEA therefore combines the specificity of an antibody-based immunoassay with the sensitivity and multiplexing capability of qPCR.

Whereas the PEA technique has essentially been utilized to develop multiplex tests (up to 96-plex), little attention has been given to assay time. However, the main limitation of PEA embodiments described in the literature is assay time, which is quite long (more than 90 min, qPCR excluded) due to multiple dilution and incubation steps in different buffers.

Procalcitonin (PCT) is a 14.5 kDa pro-hormone polypeptide which is stored, after its synthesis, in secretory granules in all cell types of the body. The rise of PCT blood levels upon bacterial, fungal or parasitic infection can routinely be measured early and specifically, which makes PCT a versatile biological marker to assist in the etiological diagnosis of an infection, help determine its severity, monitor its evolution and response to treatment, and adapt the treatment for each patient [7, 8]. It is generally accepted that for values below 0.25 μg/L a bacterial infection is excluded. A plasma dose above 0.5 μg/L indicates local infection, or even systemic bacterial infection for a dose above 2.0 μg/L. A dose of 10 μg/L or more corresponds to severe sepsis and/or septic shock [9, 10].

Here, after selecting monoclonal antibodies and designing suitable PEA probes for the detection of PCT in human plasma in a multi-well plates format, the assay time was optimized to be in line with the requirements of decentralized diagnostic testing. In an effort to simplify the PEA protocol, the use of alternative extension enzymes was also evaluated. As an initial proof of concept, a PEA protocol with a reduced time-to-result, allowing the detection of physiological amounts of PCT in very small volumes of human samples, was demonstrated.

## Material and methods

### Material

Recombinant human procalcitonin (rPCT) was developed and produced by bioMérieux SA (Lyon, France) after protein expression in prokaryotic cells, according to standard procedures [11]. rPCT was stored at -80°C in a PBS buffer containing 5% bovine serum albumin (BSA).

Whole blood specimens were obtained from healthy donors from the French National Blood Bank (*Etablissement Français du Sang Auvergne-Rhône Alpes (EFS)*, Décine Charpieu, France). Specimens were selected on the basis of a negative PCT test. Fresh blood was centrifuged for five min at 20 000 g, at 4°C, using a refrigerated microcentrifuge (Eppendorf 5424R, Montesson, France). After centrifugation, the supernatant, which corresponds to the plasma, was recovered for further analysis.

A set of PCT-positive EDTA plasma samples (leftovers) was obtained from the Saint Luc-Saint Joseph Hospital (Lyon, France) through specific supply agreements with bioMérieux SA. The PCT concentration in each of these samples was determined using a commercial IVD test (BRAHMS Vidas PCT, BioMérieux) [12].

For all biological samples, informed consent was obtained prior to any experimentation. All experiments were performed in compliance with the relevant laws and institutional guidelines and in accordance with the ethical standards of the Declaration of Helsinki. A framework agreement ("convention of session of products derived from human blood for non-therapeutic purposes") has been established between BioMérieux SA and the French National Blood Bank, whereby the latter is in charge of the ethic approval for all samples. The current agreement (EFS20-294) has been established in December 2020 for 2 years.

Monoclonal antibodies were produced by bioMérieux SA according to standard procedures, following mice immunizations with internally produced recombinant PCT [13]. These antibodies were directed either against the Calcitonin domain of the PCT molecule (monoclonal antibodies 11H4, 5G5, 4C10, 6H11 and 8F12), or against the Katacalcin domain (monoclonal antibody 11E12). A biotin-labelled version of these antibodies was also prepared for use in sandwich ELISA (Enzyme Linked Immuno-Sorbant Assay).

For PEA development, two DNA polymerases were evaluated: T4 DNA polymerase (ThermoFisher Scientific, Waltham, MS, USA) and Extaq polymerase (Takara Bio, Kusatsu, Japan). Several 3’>5’ exonucleases were also tested: exonuclease I, exonuclease VII, and exonuclease T (all from New England Biolabs, Ipswich, MS, USA).

### PEA probes

Proximity probes were prepared by covalently linking purified PCT monoclonal antibodies to HPLC-purified oligonucleotides having a C6-amin modification at the 5’ end. Synthesis, derivatization and HPLC-purification of the oligonucleotides were all performed by Integrated DNA Technologies (Coralville, USA). Antibody-oligonucleotide conjugates were prepared using commercial Thunderlink kit (Abcam, Cambridge, UK), following the manufacturer’s instructions, with a 5:1 oligonucleotide-to-antibody ratio. Conjugation quality was assessed by running conjugates on a reducing 4–12% SDS–PAGE (Thermofisher) followed by Coomassie Blue staining (ThermoFisher Scientific) as recommended by the supplier’s instructions (Abcam). The oligonucleotides comprised, from 5’ to 3’, a 18–22 nucleotides sequence for PCR primer hybridization and a 9–12 nucleotides sequence for hybridization with the second PEA probe. Sequences were generated randomly (https://molbiotools.com), taking care to minimize homeo-tracks and palindromic sequences. The PCR primers sequences had a Tm around 60°C. For the hybridization zone, a %CG between 50 and 60% was preferred.

### Sandwich ELISA

After overnight sensitization with monoclonal antibodies diluted at 5 μg/mL in PBS buffer, pH 7.4 (Euromedex, Souffelweyersheim, France), Nunc 96-well plates (ThermoFisher Scientific) were saturated for 1 h at room temperature (RT) with PBS1x-Bovine Serum Albumin 5% (BSA, Sigma-Aldrich, Saint-Louis, Mi USA). After 4 washes with PBS1x–Tween20 0.1%, plasma containing (or not) the rPCT protein (10 ng/mL) plus a biotin-labelled antibody (1 μg/mL) were added to the plate and incubated for 1 h at 37°C. The developing with PNPP substrates (ThermoFisher Scientific) was performed after 30 min of incubation at 37°C with a 1/50 000 dilution in PBS-BSA-0.5% of an alkaline phosphatase (AP)-labeled streptavidin (Jackson Immunoresearch, Ely, UK). The absorbance level (optical density, OD) was read at 405 nm (OD_405_) using an Infinite M Nano+ microplate reader (Tecan).

### PEA

The PEA reference protocol and the various buffer formulations were taken over from reference [1]. PEA was performed in specific multi-well plates (Hard-Shell 96-Well Clear shell PCR Plates, BioRad, Hercule, CA USA), in duplicate. Briefly, 4 μL of sample (PBS1x-0.1% BSA buffer with or without rPCT or human EDTA plasma) were combined with 4 μL of a mix of the two PEA conjugates (at 500 pM each) diluted in probe incubation buffer and incubated at 37°C for 1 h. After probe incubation, 96 μL of dilution buffer containing 40 mM of each dNTP were added. After a 5-min incubation step at 37°C, 96 μL of extension mix containing 26 U/mL of T4 DNA Polymerase (ThermoFisher Scientific) were added and incubated at 37°C for another 20 min, followed by a 10-min heat inactivation step at 80°C.

### Detection by qPCR

For qPCR detection, 4 μL of PEA extension products were transferred to a qPCR plate and mixed with 36 μL of qPCR mix (SYBRGREEN mix, Bio-Rad) containing 0.6 μM of each PCR primer. One-step qPCR was run with initial denaturation at 95°C for 3 min, followed by 1 sec of denaturation at 95°C and 10 sec of annealing/extension at 63°C for 40 cycles. Finally, a melt step was performed by increasing the temperature gradually from 60°C to 95°C at 0.5°C/sec.

### Statistical analysis

Means and standard deviations were calculated using Excel 2019 (Microsoft, Albuquerque NM USA).

## Results

### Raw material selection for the PEA PCT assay

PEA requires the use of two specific antibodies. To minimize assay time, antibodies must exhibit high affinity to the PCT molecule. More specifically, the on-rate constant (K-on) should be as high as possible [14]. Moreover, these two antibodies must not interfere with each other upon binding to the analyte, i.e., they must not target the same region of the protein. PCT is a small (14.5 kDa) polypeptide consisting of two domains: the calcitonin domain, located in the N-terminal region of the PCT molecule, and the katacalcin domain, which constitutes its C-terminal part. In order to limit binding interference, it was decided to select one antibody that targets calcitonin and another one that targets katacalcin. Another advantage of this approach is that PCT metabolic by-products like plasma calcitonin or katacalcin are not detected.

**Fig 1:**
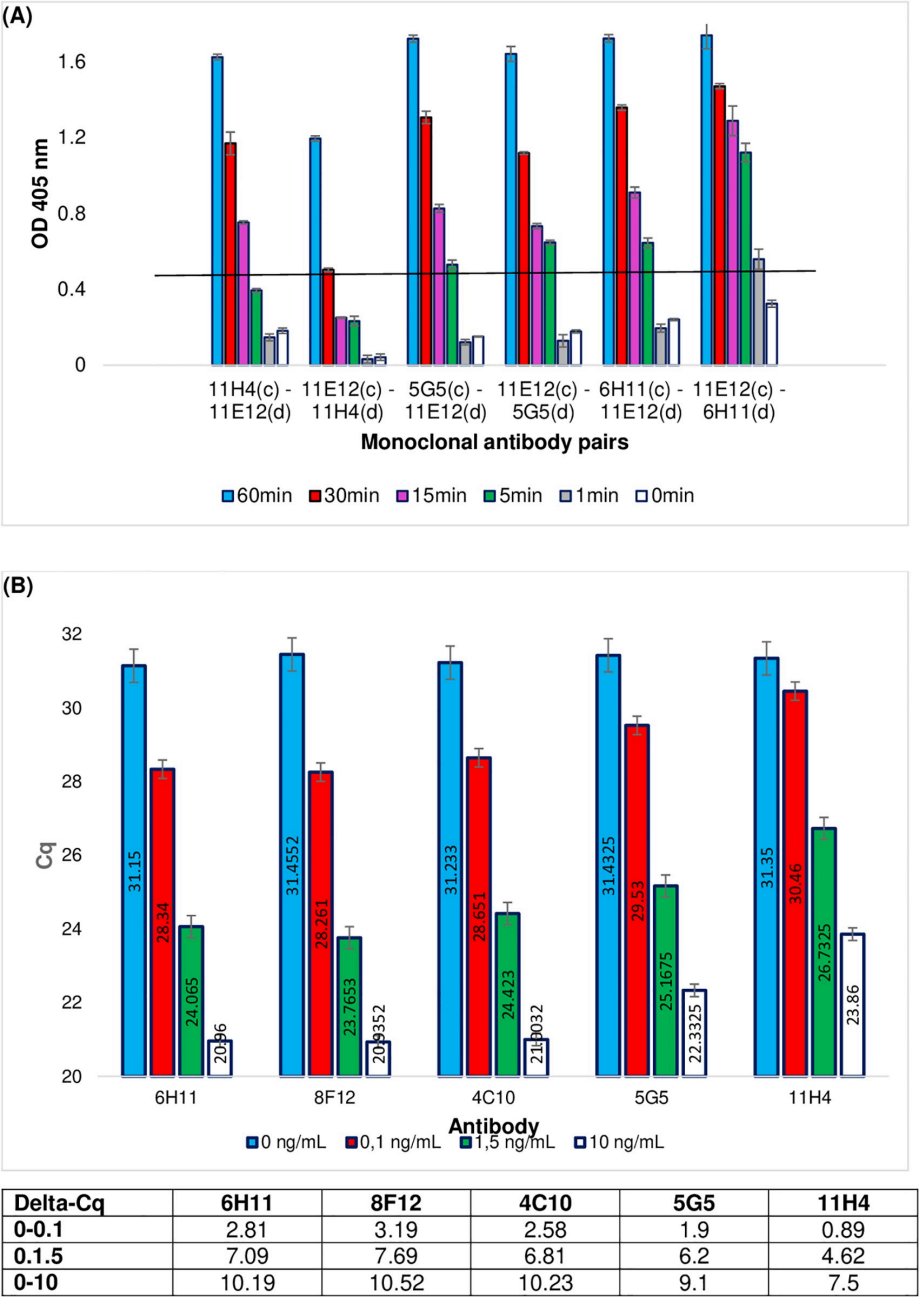
Selection of monoclonal antibody pair for PEA PCT. (A) OD at 405 nm for the different PCT antibody pairs tested in ELISA. For each pair, antibodies were tested either as capture antibody (c) or as detection antibody (d). Different incubation times were evaluated (60, 30, 15, 5, 1 and "0" minutes) for monoclonal antibodies binding to rPCT. The results correspond to the average of two duplicate experiments performed for each condition, after subtracting the blank (assay without rPCT). The black line corresponds to a positivity threshold representing 3 times the OD signal obtained on the blank. (B) PEA performed using different pairs of PCT monoclonal antibodies. Antibodies were 11E12 (present in all conditions) associated to either 6H11, 8F12, 4C10, 5G5 or 11H4. Results are given in Cq (cycle of quantification) values, which correspond to the smallest qPCR cycle number at which the fluorescence signal is significantly higher than the background. The data are also expressed in Delta-Cq (Table below the histograms) corresponding to the difference between the Cq value obtained for 0 ng/mL of rPCT and the Cq value obtained for different concentrations of rPCT (0.1, 1.5 and 10 ng/mL, respectively). The results correspond to the mean of four duplicate experiments.

PEA can be considered to be a sandwich assay. In order to choose the best pair of antibodies in terms of compatibility and affinity, several pairs of antibodies were evaluated in a sandwich ELISA format (Fig 1).

Each antibody was alternatively used either as a capture antibody (after coating on the well of the plate) or as a detection antibody (free in solution). First, these pairs were evaluated using different binding times between the antibodies and the rPCT (from 0 min to 60 min) before proceeding to the signal revelation step. Note that for the “zero” minute condition, the mix containing rPCT and detection antibody was added to the wells and immediately removed by washing. As illustrated in Fig 1A, at 60 min of incubation, which corresponds to the time dedicated to the antigen/antibody binding in the PEA reference protocol, all pairs except 11H4/11E12 performed identically, with an OD_405_ around 1.6. In contrast, for less than 5 min of incubation, the signal remained under the threshold defined as 3 times the background level (OD_405_ = 0.43), except for the 11E12-capture/6H11-detection pair [see Fig 1A: 11E12 (c) -6H11 (d)]. For 5, 15 and 30 min, the highest signal was obtained for the 11E12/6H11 pair. For 15 min of incubation, OD_405_ = 0.91 +/- 0.05 [6H11 (c)-11E12 (d)] and OD_405_ = 1.29+/-0.08 [11E12 (c)-6H11 (d)].

In order to confirm these results in the PEA format, experiments were conducted using the above antibodies (evaluated in ELISA), plus two additional antibodies called 8F12 and 4C10. All these antibodies were conjugated to oligonucleotides as described above. Fig 1B shows the Cq values obtained for four concentrations of rPCT (10 ng/mL, 1.5 ng/mL, 0.1 ng/mL and 0 ng/mL). As expected, the Cq value decreased when rPCT concentration increased. An equivalent background level (31.15< Cq_0ng/mL_<31.45) was observed for all the antibodies. However, for 0.1, 1.5 and 10 ng/mL of rPCT, the Cq was mostly lower for 6H11 and 8F12 antibodies than the other anti-calcitonin antibodies (specifically, 5G5 and 11H4). These results confirmed that monoclonal antibodies 6H11 and 8F12 (anti-calcitonin), associated to 11E12 (anti-katacalcin), gave the higher sensitivity for the three rPCT concentrations tested. Results also confirmed that the 5G5/11E12 and 11H4/11E12 pairs of antibodies were less efficient. For the 6H11-11E12 and the 8F12-11E12 pairs, Delta-Cq_0-10_ was around 1.4 higher than for the 11H4-11E12 pair. For Delta-Cq_0-0,1_, it was 2.9–3.5 higher. Consequently, 6H11 (and 8F12) and 11E12 were selected for further investigations.

Regarding probes synthesis, successive versions of oligonucleotides pairs were evaluated. Because no complete 3D-model of the PCT molecule protein structure was available, it was not possible to determine the distance between epitopes where antibodies bind. To assess whether long oligonucleotides were needed for the PEA PCT assay (as would be the case if the antibodies bind far from each other), the initial pair of oligonucleotides tested (MM1) was designed with 62–90 nucleotides (nt) sequences and a 12-mer hybridization zone. Each oligonucleotide included sequences for PCR primers hybridization in addition to the hybridization zone, separated by junk sequences. Because these junk sequences can eventually form 2D structures or facilitate unwanted hybridizations with unfavourable impact on the processivity of polymerases, some of these junk sequences were removed in the next versions of the probe (MM2 and MM4) and the hybridization zone was gradually reduced. Finally, in an effort to simplify the oligonucleotides as much as possible, the junk sequences were totally removed in the ultimate versions (MM6 to MM9), and the hybridization zone continued to be downsized. The characteristics of these successive versions, and the associated performances obtained in PEA, are illustrated in Table 1.

**Table 1:** Main characteristics of the oligonucleotide sets used for PEA.

Name of probes	Oligonucleotide sizes	Hybridation Zone (HZ) size	HZ %CG	Highest Delta-Cq_0-10_ using PEA
**MM1**	90 nt	12 nt	58.3%	3.07
	62 nt			
**MM2**	51 nt	12 nt	58.3%	3.35
	59 nt			
**MM4**	51 nt	11nt	54.5%	9.83
	59nt			
**MM6**	33 nt	11 nt	54.5%	9.98
	32 nt			
**MM7**	31 nt	10 nt	50%	**12.2**
	29 nt			
**MM9**	30 nt	9 nt	55%	**11.9**
	28 nt			

nt: nucleotides; Delta-Cq_0-10_: Cq difference between 0 and 10ng/mL of PCT.

The shorter the hybridization zone, the higher the Delta-Cq _(0–10)_. The size of the oligonucleotides seemed to have little impact since the Delta-Cq between MM4 and MM6 was similar (9.83 *vs* 9.98). In addition, the difference observed between the Delta-Cq_0-10_ of MM2 (3.35) and MM4 (9.83) can only be attributed to the size of the hybridization zone.

In conclusion, pairs of monoclonal antibodies and oligonucleotides were selected to prepare probes specifically adapted to the development of an efficient PCT PEA. The pair of PEA probes used for the rest of the study were thus prepared from the 6H11 and 11E12 monoclonal antibodies conjugated to the MM7 oligonucleotides.

### PEA assay time reduction

In order to reduce the duration of the assay while maintaining a high level of performance, a gradual decrease in incubation times was assessed for each step of the assay. This was first conducted separately and then evaluated in a single merged protocol.

**Fig 2:**
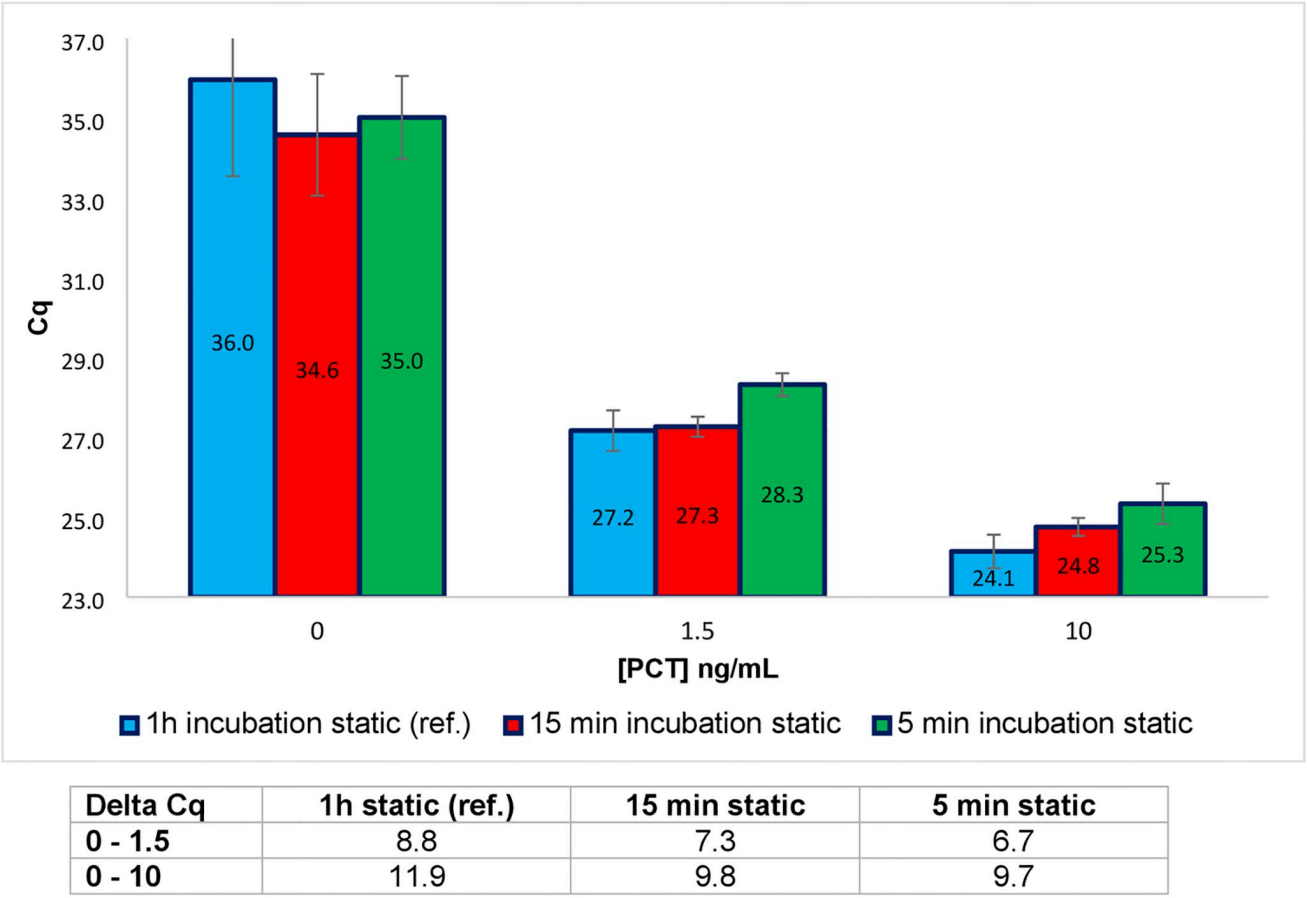
PEA performed for different durations of antigen/antibody binding, for three concentrations of rPCT. The time of incubation between the rPCT and the PEA probes was progressively decreased from 1h (reference test, ref.) down to 5 minutes. Incubation was performed at 37°C under static conditions (no agitation). The results, given in Cq, correspond to the mean of a minimum of three duplicate experiments. The data are also expressed in Delta-Cq (Table below the histograms) corresponding to the difference between the Cq value obtained for 0 ng/mL of rPCT and the Cq value obtained for different concentrations of rPCT.

Conventionally, in the reference protocol, the time required for the binding of the antigen to the PEA conjugates is about one hour. Different incubation times, including 15 min and 5 min, were tested for three concentration of rPCT (0, 1.5 and 10 ng/mL) diluted in PBS1x-BSA. These rPCT concentrations correspond to concentrations of PCT found in patients with systemic bacterial infections (1.5 ng/mL) or severe sepsis (10 ng/mL). No agitation of the plates was provided during incubation, which took place in static conditions. As illustrated in Fig 2, using the pair of PEA probes previously selected, the Cq value obtained with 1 h or 15 min of incubation was not significantly different for the two concentrations of rPCT. However, at 5 min of incubation a slight but significant loss of sensitivity was observed for 1.5 and 10 ng/mL of rPCT.

Results expressed in Delta-Cq also showed that for 1.5 ng/mL of rPCT, with 15 min or 5 min of incubation, the differences of Delta-Cq (Delta [Delta-Cq]) for 1h of incubation was 1.5 and 2.1, respectively. For 10 ng/mL of rPCT, the Delta [Delta-Cq] was 2.1 and 2.2, respectively.

**Fig 3:**
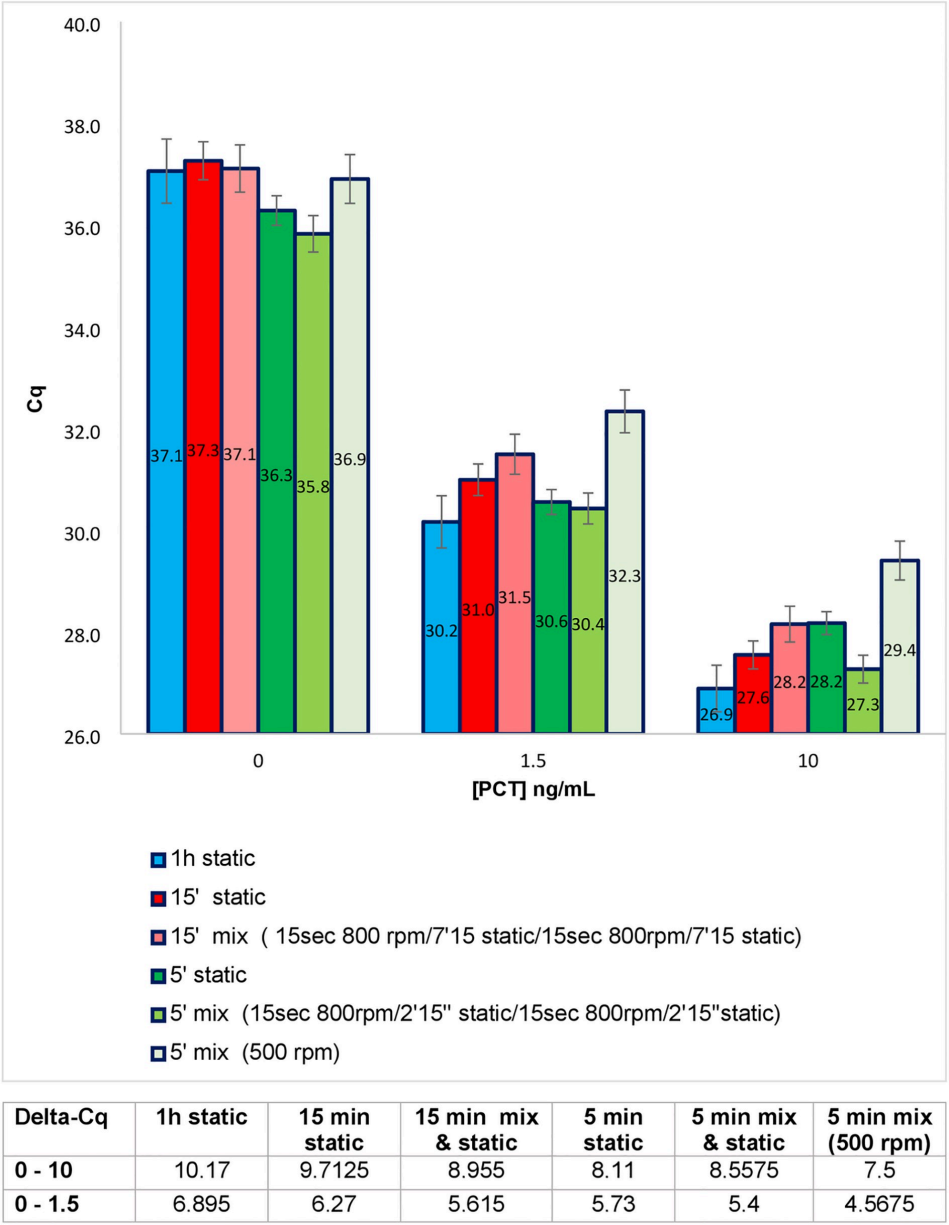
Effect of plate agitation on PEA performed for different durations of antigen/antibody binding for three concentrations of rPCT. The incubation time between the rPCT and the PEA probes was progressively decreased from 1h (reference test) down to 5 minutes. The incubation was performed at 37°C under static conditions (static) or with agitation of the multi-well plate in a thermomixer. Two alternative mixing conditions were tested: one with continuous agitation at 500 rpm, the other with phases of agitation at 800 rpm alternating with phases without agitation (static). The results, given in Cq values, correspond to the mean of a minimum of three duplicate experiments. The data are also expressed in Delta-Cq (Table below the histograms) corresponding to the difference between the Cq value obtained for 0 ng/mL of rPCT and the Cq value obtained for different concentrations of rPCT.

In order to improve the association kinetics over incubation times of 5 or 15 minutes, the binding step was performed with plate shaking using an Eppendorf thermomixer heated to 37°C. This agitation was either continuous (500 rounds per minute, rpm) or intermittent (agitation at 800 rpm followed by no agitation, repeated twice). The results obtained are summarized in Fig 3. The reference corresponded to a binding step conducted for 1 h on the same thermomixer but without shaking. The graph shows that 15 min of incubation with intermittent agitation did not bring the Cq to the same level as that observed for the reference. For 5 min of incubation, it was possible to decrease the Cq by adding intermittent agitation. This effect was more significant for the highest tested concentration of PCT. Interestingly, 5 min of incubation without agitation also led to Cq values equivalent to the reference, but only for the lowest tested concentration of PCT. In an unexpected way, continuous agitation at 500 rpm dramatically increased the Cq. Based on Delta-Cq_0-10_, 1h of incubation caused the highest difference (Delta-Cq_0-10_ = 10.2), closely followed by conditions with 15 min of incubation (8.9 or 9.7 when mixing was added). A 5 min incubation time corresponded to a lower Delta-Cq_0-10_, with values around 8.3. The lowest Delta-Cq_0-10_ was obtained with 5 min of incubation with continuous mixing (Delta-Cq_0-10_ = 7.5). For Delta-Cq_0-1.5_ the trend was quite identical, with lowest performance associated to 5 min of incubation with continuous agitation. Interestingly, for Delta-Cq_1.5–10_ all the values were equivalent (between 2.9 and 3.3), except for 5 min of incubation without mixing, where Delta-Cq_1.5–10_ was 2.4.

In summary, it was possible to decrease the incubation time down to 15 min without significantly affecting the performance of the assay. For 5 minutes of hybridization, it was necessary to shake the plate on and off to have PEA performances approaching those obtained with the reference conditions.

**Fig 4:**
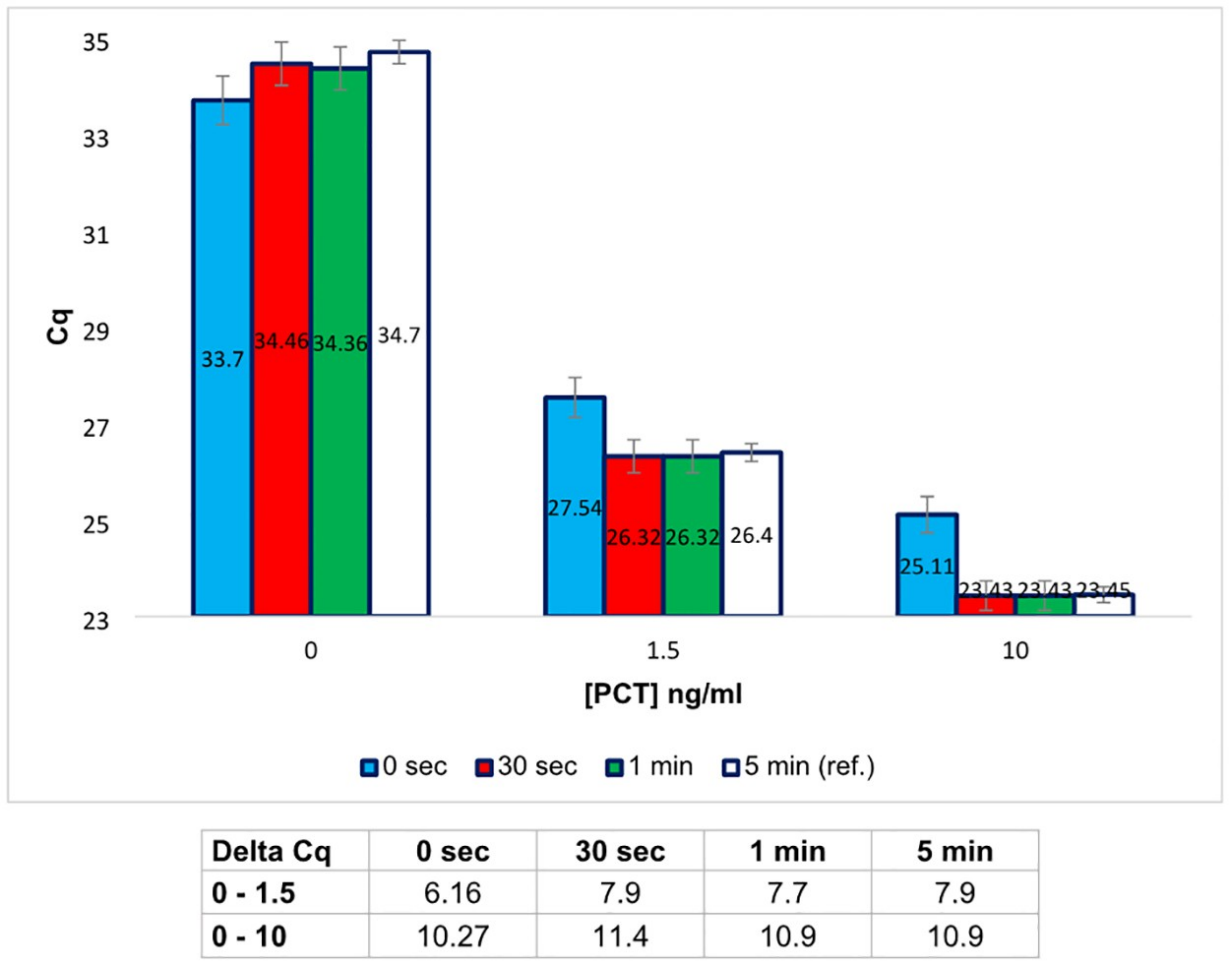
PEA performed for different durations of incubation after the dilution step, for three concentrations of rPCT. The incubation time after dilution of the binding product was progressively decreased from 5 min (reference test, ref.) down to 30 sec. The results, given in Cq values, correspond to the mean of a minimum of three duplicate experiments. The data are also expressed in Delta-Cq (Table below the histograms) corresponding to the difference between the Cq value obtained for 0 ng/mL of rPCT and the Cq value obtained for different concentrations of rPCT.

Another step of the PEA protocol that can potentially be reduced is the 5 min incubation period taking place just after the dilution that follows the binding of the PEA probes to the PCT. As illustrated by the graph presented on Fig 4, a decrease to 30 sec of the incubation time did not significantly affect the Cq value. However, it was not possible to completely remove this step because a slight but significant reduction of the signal was then observed.

**Fig 5:**
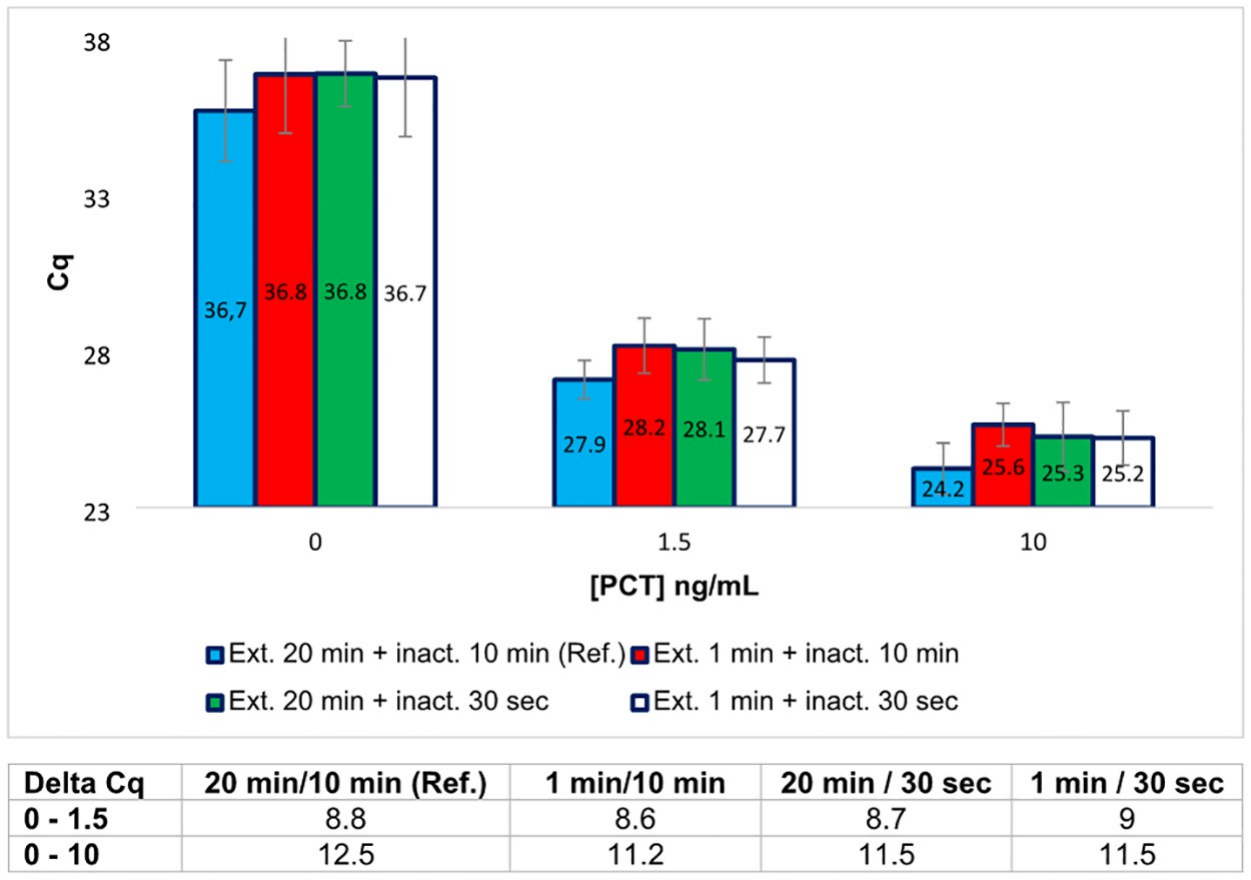
PEA performed at different extension times and different enzyme inactivation times, for three concentrations of rPCT. The time of extension (Ext.) of the duplex by T4 DNA polymerase was progressively decreased from 20 min (reference test, ref.) down to 1 min. In parallel, the time of inactivation of the enzyme (inact.) was reduced from 10 min (reference, ref.) down to 30 sec at 80°C. The results, given in Cq values, correspond to the mean of a minimum of three duplicate experiments. The data are also expressed in Delta-Cq values (Table below the histograms) corresponding to the difference between the Cq value obtained for 0 ng/mL of rPCT and the Cq value obtained for different concentrations of rPCT.

The extension step triggered by adding T4 DNA polymerase is crucial because it promotes complementary strand synthesis after hybridization of the two PEA probes at their 3’-ends. It also promotes background noise reduction by hydrolysing the unmatched PEA probes thanks to its 3’>5’ exonuclease activity [15, 16]. After an extension step of 20 min at 37°C, the enzyme must be heat-inactivated at 80°C during 10 min. To assess whether it was possible to reduce the extension time and the inactivation time without impacting the performance of the assay, several experiments were conducted successively. First, the duration of the extension step was reduced from 20 min to 1 min. As shown in Fig 5, no significant difference was observed between the two conditions tested. Second, when the T4 DNA polymerase was inactivated for 30 sec, instead of 10 min at 80°C, using the 20 min-extension protocol, no difference was observed. Finally, when a reduced time of extension step (1 min at 37°C) was combined with a short time of inactivation, no significant difference with the reference method was observed.

**Fig 6:**
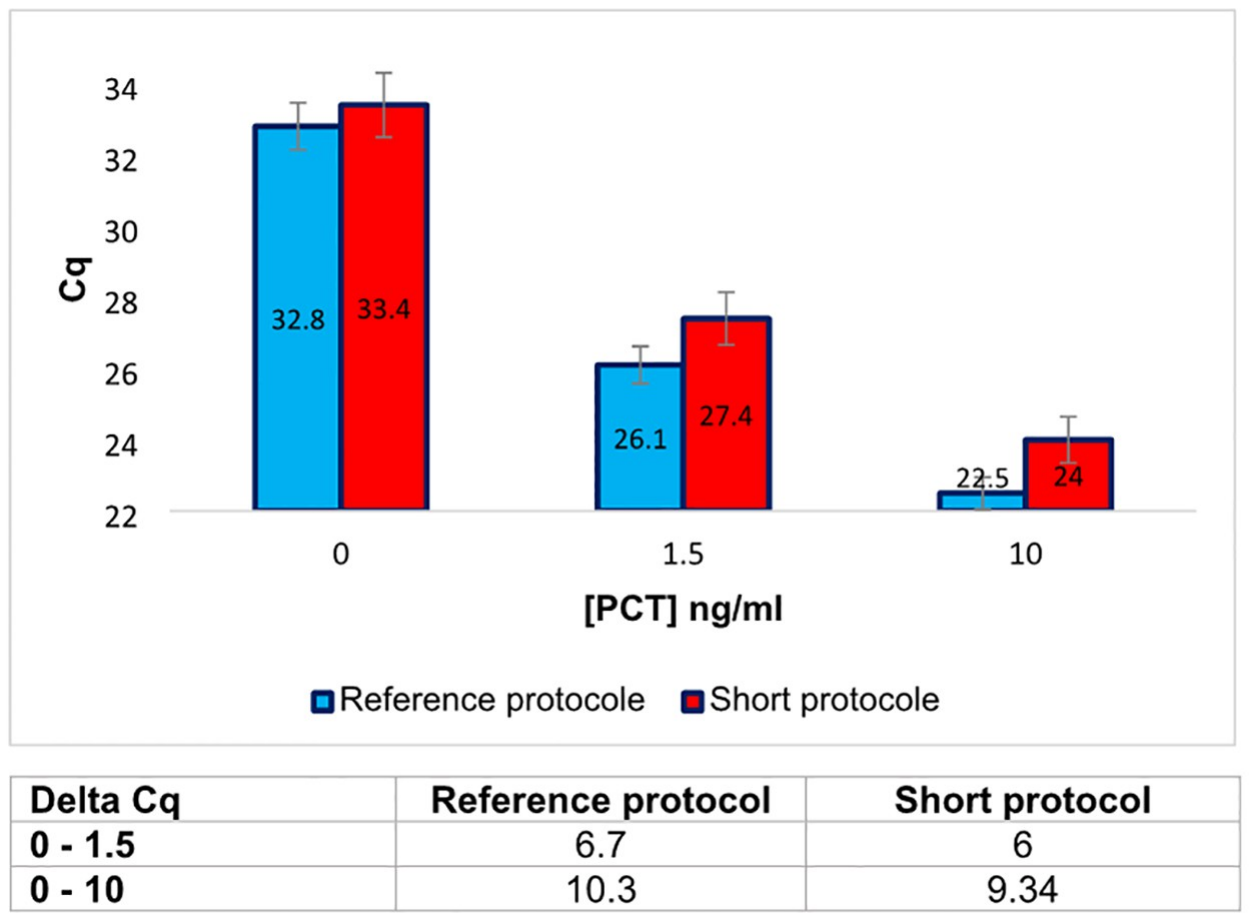
Short PEA protocol implementation in its full form on rPCT spiked in PBS-BSA buffer. The short protocol was compared in terms of Cq values with the reference protocol, on three rPCT concentrations. The results, given in Cq, correspond to the mean of a minimum of three duplicate experiments. The data are also expressed in Delta-Cq (Table below the histograms) corresponding to the difference between the Cq value obtained for 0 ng/mL of rPCT and the Cq value obtained for different concentrations of rPCT.

The whole shortened protocol thus corresponded to 5 min of antigen and PEA probe binding (with intermittent agitation), followed by 30 sec of incubation after the dilution step, and 1 min + 30 sec for T4 DNA polymerase extension and inactivation, respectively, for a total incubation time of 7 minutes. This 7 min protocol was tested in multi-well plates on PBS1x-BSA buffer spiked with rPCT at different concentrations. Results are presented in Fig 6. Except for 10 ng/mL of rPCT where a small gap of 1.5 Cq was observed, there was no significant difference between the short protocol and the reference protocol for the detection of rPCT.

Thus it is possible to drastically reduce the time needed for PEA without significantly affecting the performance of the assay for PCT detection.

### Alternative enzymes to T4 DNA polymerase

**Fig 7:**
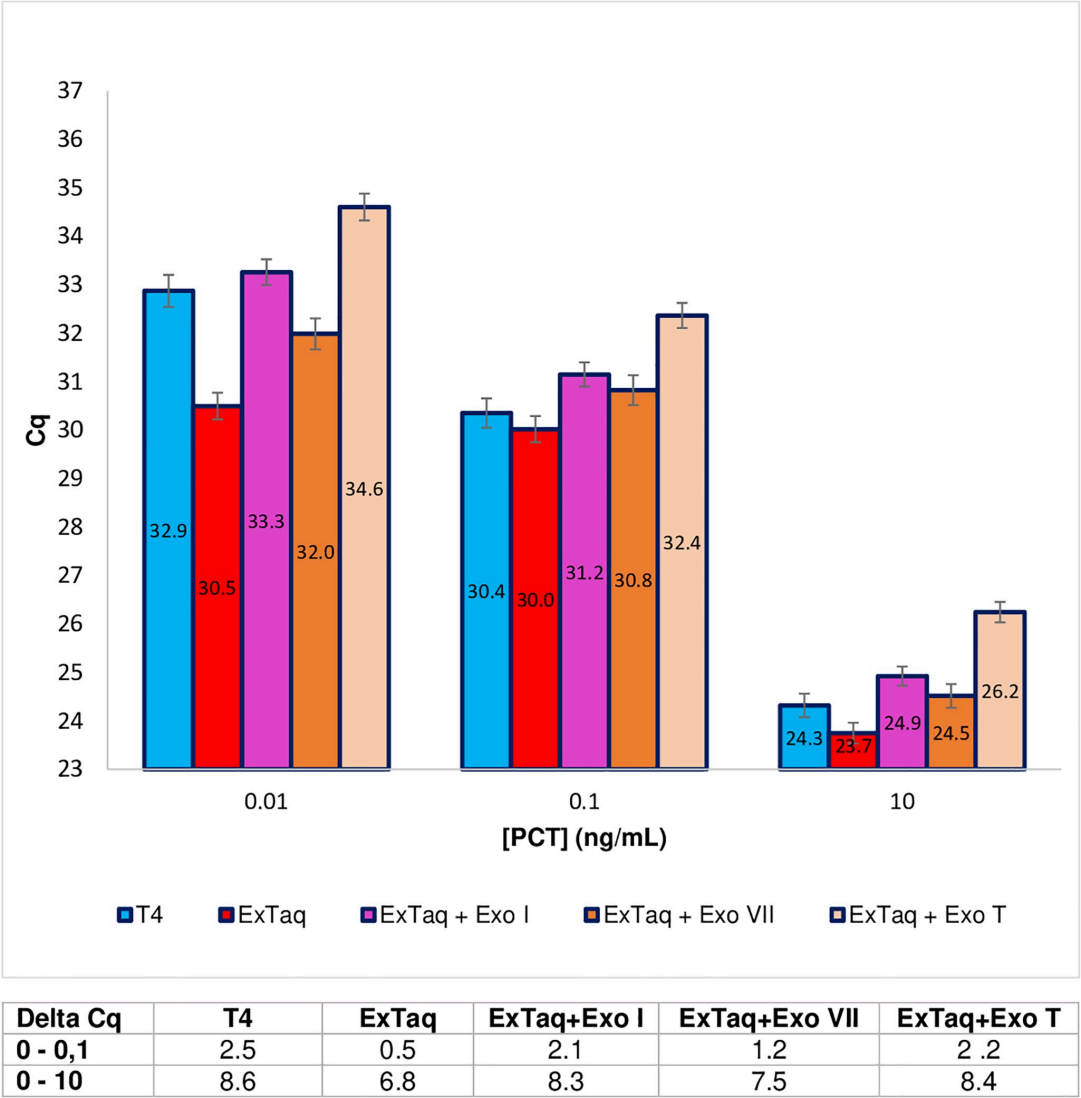
Comparison of different enzymes or enzyme combinations for PEA extension step (short protocol), for three concentrations of rPCT. T4: T4 DNA polymerase; Extaq: Extaq polymerase; Exo I: exonuclease I; Exo VII: exonuclease VII; Exo T: exonuclease T. The results, given in Cq, correspond to the mean of a minimum of three duplicate experiments. The data are also expressed in Delta-Cq (Table below the histograms) corresponding to the difference between the Cq value obtained for 0 ng/mL of rPCT and the Cq value obtained for different concentrations of rPCT.

It has been previously demonstrated that 3’>5’ exonuclease activity exhibited by the T4 DNA polymerase reduced nonspecific background and increased PEA sensitivity [4]. To assess whether it is possible to replace T4 DNA polymerase by another DNA polymerase combining comparable exonuclease activity and higher processivity, a thermostable DNA polymerase having these characteristics was evaluated (Extaq, Takara). The challenge consisted in eliminating the extension step, by simply adding Extaq to the extension buffer, mixing and then immediately proceeding to the PCR amplification step. As shown in Fig 7, the use of Extaq instead of T4 resulted in increased background and decreased sensitivity of PEA. Indeed, the Delta-Cq_0-0.1_ was 0.5 for the Extaq and 2.5 for the T4 DNA polymerase. To test the hypothesis that these unsatisfactory results were due to a lower than expected exonuclease activity of Extaq, different exogenous exonucleases (Exonuclease I, Exonuclease VII and Exonuclease T) were evaluated in combination with Extaq. All of these exonucleases, with the exception of Exonuclease T, used in combination with Extaq resulted in restored sensitivity and improved signal-to-noise levels. Exonuclease I and Exonuclease T showed significant improvement in background. However, in terms of Delta-Cq, the performance was similar to that obtained with T4 DNA polymerase: for example, Delta-Cq _0–10_ was 8.6 for T4 DNA polymerase on its own and 8.3 and 8.4 for Extaq combined with Exonuclease I and Exonuclease T, respectively.

In conclusion, the T4 DNA polymerase can advantageously be replaced by a thermostable DNA polymerase, in terms of background noise reduction, provided that this enzyme exhibits a strong 3>5’ exonuclease activity.

### Implementation of the short protocol for PCT detection in human samples

It was crucial to confirm that the fast PEA protocol presented henceforth could be applied to real patient plasma samples while maintaining adequate performance. To validate the short PEA protocol on real human samples (as opposed to synthetic samples prepared by spiking rPCT expressed in *E*. *Coli* into PBS-BSA buffer), the protocol was applied to a variety of human plasmas from several patients having a range of naturel PCT concentrations from 0.08 ng/mL to 48 ng/mL. For this experiment, the probe incubation buffer was complemented by 0.25 mg/mL of purified mouse antibody (Meridian), to mitigate any potential Human Anti-Mouse Antibodies (HAMA) interference [17].

Plasma specimens were obtained from patients with PCT concentrations ranging from the limit of sensitivity required to diagnose bacterial infection (i.e., 0.1 ng/mL) to exceptionally high values (>20 ng/mL), outside the concentration ranges generally observed. A few samples with PCT concentrations above 0.5 ng/mL, indicative of a proven, possibly severe infection, were also tested. These samples were mainly taken from men (5 men and 2 women) and the average age was 66 ± 19. We did not have access to the patients’ medical records. Two PCT negative plasma were also included in this experiment.

**Fig 8:**
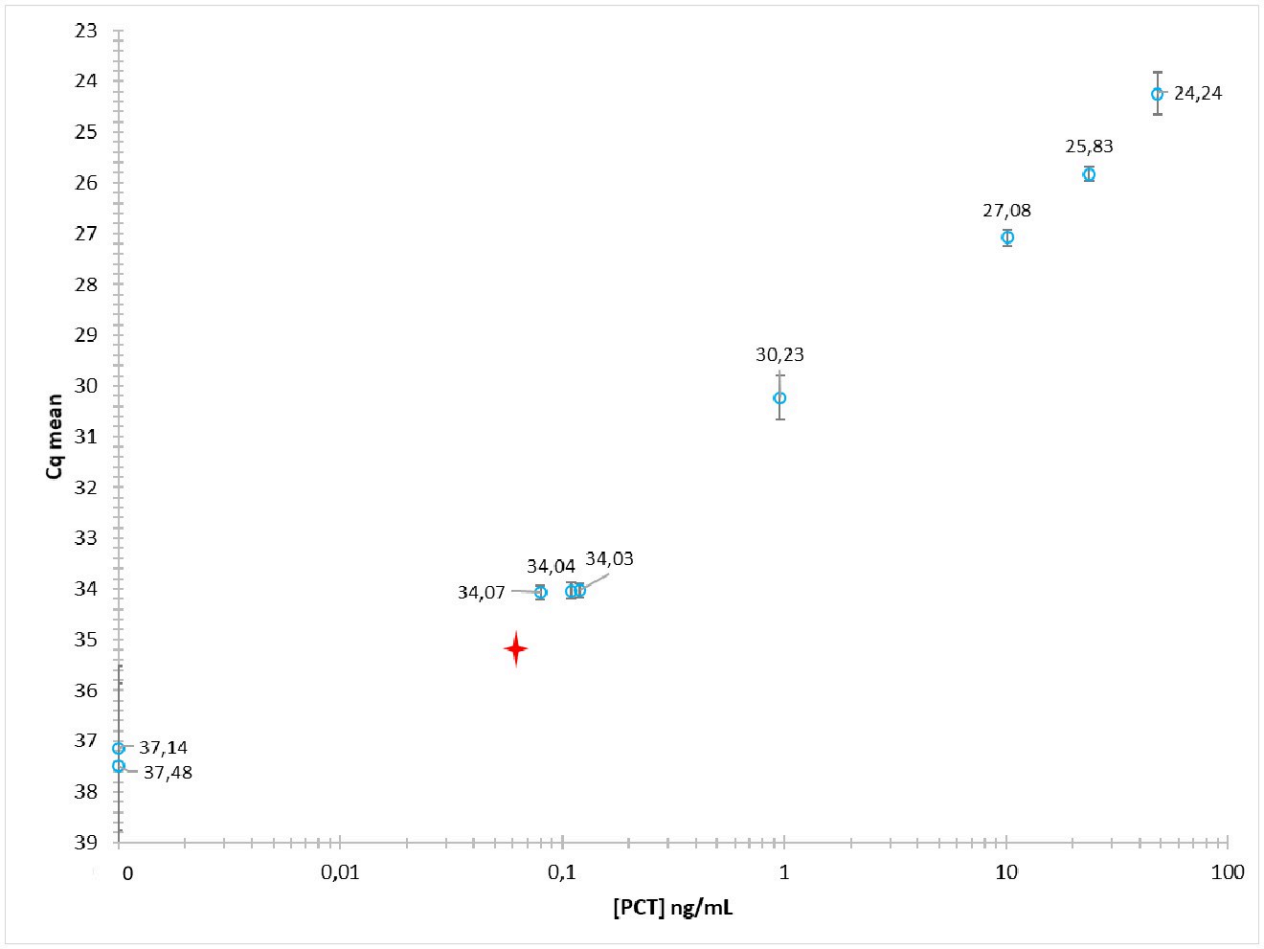
Short PEA protocol implemented on human plasma specimens containing different concentrations of PCT. The results, given in Cq values, correspond to the mean of two or three duplicate experiments. The red star corresponds to an estimation of the LoD.

The results, which correspond to the average of independent experiments performed in duplicate, are shown in Fig 8. It was thus possible, using the short PEA protocol developed here, to outline a dose-response curve for different concentrations of PCT in plasma samples from human patients. At 0.08 ng/mL of PCT, the Cq signal is significantly different from the background (error bars correspond to 2 times the standard deviation).

The Limit of Detection (LoD) was estimated to be 0.063 ng/mL (Cq = 35.21). However, to achieve a most robust estimate of the LoD, it should be determined from samples tested repeatedly, usually 10–20 times [18].

## Discussion

The discovery and application of blood biomarkers play a central role in clinical medicine. The ability to identify disease-related physiological processes from a blood sample contributes to effective clinical diagnosis. While analyses at the transcript level often require invasive sampling or biopsy, biomarker analysis can allow for sufficiently sensitive diagnosis, prognosis or stratification of patients by simple blood sampling.

In this regard, the sandwich ELISA technique is very popular in clinical practice [16]. These assays are most often performed using an immobilized capture antibody, while the captured target protein is revealed by binding to second antibodies conjugated to enzymes, providing a detectable signal by enzymatic conversion of colorimetric, fluorescent or chemiluminescent substrate molecules. One major drawback of ELISA is that it is not well suited to multiplex measurements due to cross-binding of antibodies. This problem increases exponentially with the degree of multiplexing.

Immuno-PCR (i-PCR), first described in 1992 [19], involves a specific monoclonal antibody to which a linear DNA tag is bound, and a second specific antibody immobilized on microtiter-plate wells or magnetic beads. Amplification of the DNA tag by PCR after formation of the immune-sandwich generates exquisite levels of signal. While the first versions of i-PCR required time-consuming and labour-intensive post-PCR analysis, the use of qPCR has not only simplified i-PCR protocols, but also increased the dynamic range of the assay [20, 21]. Today, i-PCR is often considered to be a robust method that offers the specificity and sensitivity required for many clinical applications, including the detection of biomarkers of diagnostic interest [22, 23]. The main drawback of i-PCR is its non-homogeneous nature, which requires numerous wash steps to ensure minimal background.

Proximity assays, developed more recently, largely address this problem. PLA was first documented in 2002 [2]. PLA uses probes that are usually obtained by covalent attachment of two separate oligonucleotides to two separate antibodies specific for the biomarker to be detected. Briefly, the assay workflow consists in binding the antibodies to the biomarker via their epitopes, followed by a ligation step that generates amplifiable DNA templates, and finally signal generation that can be based on different readout formats. PLA can be performed in solution as a homogeneous assay, i.e. without requiring a solid phase, which has the advantage of minimizing operator intervention, negating the need for washes and thus allowing for shorter assay times. Like i-PCR, PLA assays can also be configured in a solid-phase format using an immobilized capture antibody, with two proximity probes binding to the captured target molecules [24, 25], an approach that may be more suitable for detecting proteins directly from biofluids such as blood or feces. Multiplexed PLAs have also been developed [26]. The main drawback of PLA is related to the use of DNA ligase, an enzyme whose performance is highly dependent on the incubation temperature and very sensitive to inhibitors present in the biological samples to be analysed.

PEA represents an alternative to PLA and was initially developed to mitigate the fact that proximity probes joined by a DNA ligase suffer from recovery loss in complex biological fluids [1]. The main difference between the two methods is that in PEA the ligation event is replaced by a DNA polymerisation step. Furthermore, judicious choice of DNA polymerase allows minimisation of background noise and so improves the sensitivity of the assay [5]. PEA is readily amenable to multiplexing [4, 5] and features the same advantages as PLA, including very low sample consumption, wash-free workflow, and high sensitivity (typically around 10 pg/mL) and specificity. For maximal sensitivity, PEA requires the use of DNA polymerases with 3’>5’ exonuclease activity as these reduce background noise by degrading non-proximal DNA strands.

Other assays inspired by proximity assays, such as the Antibody Detection by Agglutination-PCR (ADAP), have been developed to detect antibodies (that can be considered as a separate class of biomarkers) and have been claimed to be simple, ultrasensitive and multiplexable [27, 28].

The main objective of the work presented here was to utilize the PEA for the low-plex detection of markers of medical interest in human samples, and more specifically in the context of decentralized testing, where ease of implementation and short time-to-results are key. More precisely, the aim was to establish a proof-of-principle for a modified PEA protocol which would be more compatible with the short assay times that are required in the context of point-of-care clinical diagnostics (generally less than 30 min). This modified PEA protocol was thus designed to be simpler and faster than the reference PEA protocol, without seeking to support high levels of multiplexing.

The biomarker of interest to us was PCT, which is used in clinical practice for improved patient management in case of suspected bacterial infection.

It has been shown that PEA performance depends on the affinity of the antibody pair for their respective targets and also on the design of the oligonucleotides making up the PEA probes. Previous studies conducted by Lundberg et al. [1] showed that PEA probes with a 9 nt hybridization zone gave the highest signal-to-noise level among a variety of probes with hybridization zones of different lengths. The outcome of the investigation presented here was identical, i.e., the size of the hybridization zone was found to have a major importance on PEA sensitivity, with the ideal size being 9–10 nt long.

It has been demonstrated that it is possible to drastically reduce, by more than 13.5 times, from 95 min to 7 min, the PEA assay time without significantly affecting the performance of the assay for PCT detection and quantification. The time dedicated to PCT/PEA probes binding was significantly reduced (from 60 to 5 min). The selection of monoclonal antibody pairs showing high affinity to PCT partially explain these data. To upgrade the assay to a multiplex assay (whereby additional biomarkers are detected concomitantly with PCT), it will be necessary to select antibody pairs with high affinity for the additional target(s). For instance, CRP (C-Reactive Protein), a biomarker of inflammation, could be associated to PCT to increase the medical value of the assay for infection diagnostics. In a joint investigation which has been submitted for publication, an alternative pair of antibodies was selected for the development of an internal control to PCT PEA. It was shown that both markers (internal control and PCT) could be detected concomitantly with the modified PEA protocol without affecting assay performance.

It has also been demonstrated that intermittent shaking was preferable over continuous agitation, supposedly because the shearing forces caused by constant shaking did not promote the interaction of ligands to their target. The strength of PEA is that it combines antibody-based biorecognition with PCR-based amplification of the signal, which provides remarkable level of sensitivity and specificity. Signal lost during the shortened time of hybridization may probably be compensated by a gain in signal amplification by PCR.

The incubation time dedicated to elongation by T4 DNA polymerase was also reduced, in consideration of the short complementary stand to be synthesized (up to 21 nucleotides) and an expected polymerase rate of 250–400 nucleotides/sec, the complementary stand that it has synthesized was up to 21 nucleotides. Even if the enzyme is considered to have low processivity in the absence of phage T4 accessory proteins, 30 sec seems to be sufficient for complete complementary strand synthesis and background noise reduction [15, 29].

The 30 seconds of denaturation at 80°C applied after the T4 elongation step appeared to be sufficient to neutralize the enzyme activity, since the results obtained were comparable to those obtained with 10 minutes of denaturation at 80°C. A complementary study previously conducted in our laboratory and not presented here, showed that T4 DNA polymerase was very sensitive to heat and could be rapidly inhibited after a few tens of seconds at temperatures between 70 and 80°C.

To reduce the number of steps needed and consequently the time required to perform the complete PEA protocol, alternative enzymes such as thermostable polymerases with 3’>5’ activity were evaluated. The objective was to perform the PEA extension and PCR amplification steps using the same enzyme. The hypothesis was that the residual activity of Extaq at 37°C was sufficient to synthesize the 19 and 21 complementary nucleotides of the duplex. Unfortunately, the performance obtained using Extaq was lower than expected. However, the addition of exogenous exonuclease led to PEA performance close to that obtained with T4 DNA polymerase, with an additional reduction in background noise. This is further evidence of the crucial role of the 3’>5’ exonuclease activity of the elongation enzyme on the performance and sensitivity of PEA. In a previous study, Assarsson et al. [5] have tried to simplify PEA protocol steps without increasing the background noise. They used alternative thermostable enzymes to T4 DNA polymerase, such as Pfu or Pwo polymerases. Their hypothesis was that these enzymes have low or no activity at room temperature, which would make it possible to combine all PEA reagents at the same time, with low background noise. The Pwo Hypernova polymerase was the thermostable enzyme that best met the hypothesis.

The choice of the most appropriate enzyme is one illustration of the many avenues that can be explored to improve the sensitivity of our fast PEA protocol. It would also be worthwhile to optimize the formulations of the various PEA buffers (in terms of ionic strength, salt, adjuvants, blocking agents, etc), or the quality and concentration of PEA probes, among other factors.

A large number of fully automated immunoassays have become commercially available over the past decade, based on various assay formats including enzymatic, luminescent, fluorescent and turbidimetric methods. As the clinical management of patients with severe infections or sepsis is eminently critical and time-dependent, the availability of fully automated PCT immunoassays with high throughput, short time-to-result, low sample volume, and reasonable cost, is essential [30].

Results of a multi-center evaluation conducted by Dipalo et al. on several commercial systems [31] suggested that the different solutions tested exhibited comparable performance for the diagnostic thresholds of 2.0 and 10 ng/mL of PCT, which reflect moderate and high risk of progression to severe infection and sepsis. The functional sensitivity was generally between 0.01 and 0.05 ng/mL [32].

Nevertheless, Lippi et al. [30] showed considerably lower LoB (limit of blank), LoD (limit of detection) and functional sensitivity values (all ≤ 0.003 ng/mL) for recently commercialized system, better than other fully automated techniques.

For the Vidas BRAHMS PCT kit, used here as reference diagnostic kit, the analytical limit of detection was specified as 0.05 ng/mL and the functional limit of detection 0.09 ng/mL (https://www.biomerieux-diagnostics.com/vidasr-brahms-pct).

Overall, the performance of the assay presented in the present paper is competitive with that of the majority of commercial solutions and should allow for adequate quantification of PCT at the various diagnostic thresholds that reflect the different stages of a bacterial infection, from local to systemic.

In the future, it would be useful to combine this fast PEA method with an ultrafast PCR amplification of a few minutes on micro-volumes of PEA reaction mixture [33] in order to further decrease the assay time to a level fully compatible with medical decision-making at the point-of-care. In this regard, the automation and integration of the protocol into a low-cost, compact, fully-automated, sample-to-result, low-plex version of the assay based on a microfluidic cartridge and associated instrumentation could bring significant added value to the decentralized testing of PCT-based diagnosis, such as sepsis and Lower Respiratory Tract Infections.

In conclusion, a fast assay based on PEA technology was demonstrated that enables quantification of PCT over a large range of concentrations and with low limit of detection (less than 0.1 ng/mL) in human plasma. Simultaneous direct capture and detection with two monoclonal PCT antibodies imparts high specificity to the assay, paving the way to multiplexing with other analytes. The method would benefit from further improvements (particularly when the expected concentrations are very low, between 0.05 and 0.1 ng/mL of PCT) and further evaluation for possible utilization in diagnostic applications, most interestingly in the context of a point of care solution.

## Supporting information

S1 Fig PEA conjugate control on SDS-PAGE. 3μg of each conjugate are loaded on SDS-PAGE before staining with Coomassie blue. PEAp1, PEAp2: PCT conjugates; PEA T+: Thunderlink kit control conjugate; M: molecular weight ladder (GeneRuler DNA, Thermo Scientific).
(PDF)

S2 Fig PEA oligonucleotide sequences. In red: hybridization zone; bold underlined: PCR primer sequences.
(PDF)

S1 Table Details of PCT-positive EDTA plasma. M: male; F: female.
(PDF)
