# Supplementary figures and images for: Procalcitonin detection in human plasma specimens using a fast version of proximity extension assay
Source: PLoS One. 2023 Feb 16;18(2):e0281157. doi: 10.1371/journal.pone.0281157 (PMC9934411; doi:10.1371/journal.pone.0281157)

| PEAp1 | PEAp2 | PEA T+ | M |
|-------|-------|--------|---|
|-------|-------|--------|---|

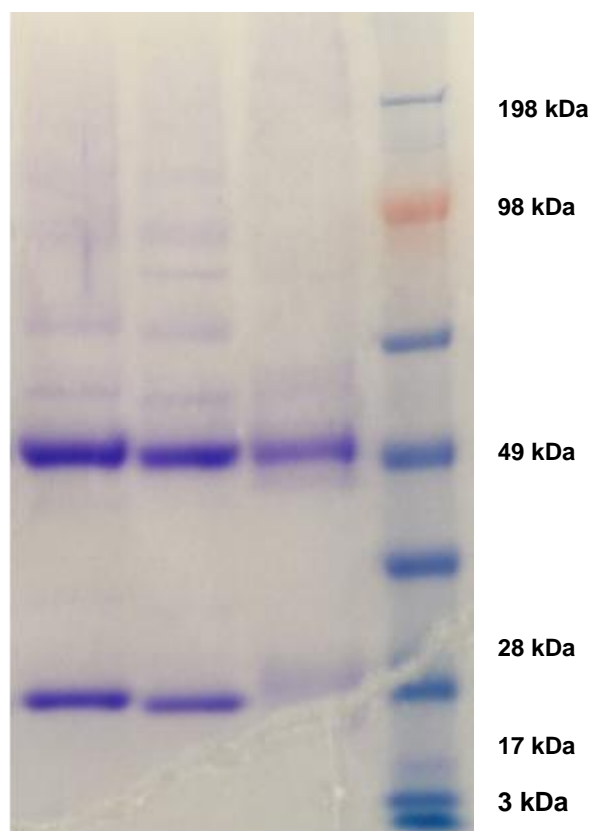

Supplement: S1 Fig — 3μg of each conjugate are loaded on SDS-PAGE before staining with Coomassie blue. PEAp1, PEAp2: PCT conjugates; PEA T+: Thunderlink kit control conjugate; M: molecular weight ladder (GeneRuler DNA, Thermo Scientific). (PDF) [file pone.0281157.s001.pdf]
