# Supplementary material for: Procalcitonin detection in human plasma specimens using a fast version of proximity extension assay
Source: PLoS One. 2023 Feb 16;18(2):e0281157. doi: 10.1371/journal.pone.0281157 (PMC9934411; doi:10.1371/journal.pone.0281157)
Supplement: S2 Fig — In red: hybridization zone; bold underlined: PCR primer sequences. (PDF) [file pone.0281157.s002.pdf]

Oligonucleotide PEAp1 : T**ATCTGTCATGGAGGAACGAG**GACGACTTCC (n=31)

Oligonucleotide PEAp2 : **GCACTATCAGCGTGCAACG**GAAGTCGTCC (n=29)
