# Supplementary material for: Procalcitonin detection in human plasma specimens using a fast version of proximity extension assay
Source: PLoS One. 2023 Feb 16;18(2):e0281157. doi: 10.1371/journal.pone.0281157 (PMC9934411; doi:10.1371/journal.pone.0281157)
Supplement: S1 Table — M: male; F: female. (PDF) [file pone.0281157.s003.pdf]

Plasma EDTA	PCT [ng/mL]	Date of sampling	Age (years)	Sex
**7**	48.2	FEV 2018	85	F
**6**	23.66	FEV 2018	75	M
**5**	10.12	FEV 2018	45	M
**4**	0.96	MAR 2018	63	M
**3**	0.12	FEV 2018	75	M
**2**	0.11	MAR 2018	82	M
**1**	0.08	FEV 2018	37	F
0 *(a)*	*Not detected*	*2020*	*40*	*M*
0 *(b)*	*Not detected*	*2020*	*62*	*F*
